# Elevated plasma p-tau231 is associated with reduced generalization and medial temporal lobe dynamic network flexibility among healthy older African Americans

**DOI:** 10.1186/s13195-024-01619-0

**Published:** 2024-11-22

**Authors:** Miray Budak, Bernadette A. Fausto, Zuzanna Osiecka, Mustafa Sheikh, Robert Perna, Nicholas Ashton, Kaj Blennow, Henrik Zetterberg, Patricia Fitzgerald-Bocarsly, Mark A. Gluck

**Affiliations:** 1 Center for Molecular & Behavioral Neuroscience, Rutgers University–Newark, 197 University Avenue, Suite 209, Newark, NJ 07102 USA; 2 Department of Psychiatry and Neurochemistry, Institute of Neuroscience and Physiology, The Sahlgrenska Academy at the University of Gothenburg, Wallinsgatan 6, Mölndal, Gothenburg, 431 41 Sweden; 3 Clinical Neurochemistry Laboratory, Sahlgrenska University Hospital, Box 100, Mölndal, Gothenburg, 405 30 Sweden; 4 Department of Neurodegenerative Disease, UCL Institute of Neurology, Queen Square, London, WC1N 3BG UK; 5 UK Dementia Research Institute at UCL, 6th Floor, Maple House, Tottenham Ct Rd, London, W1T 7NF UK; 6 Hong Kong Center for Neurodegenerative Diseases, Clear Water Bay, Units 1501- 1502, 1512-1518, 15/F Building 17W, 17 Science Park W Ave, Science Park, Hong Kong, China; 7 Wisconsin Alzheimer’s Disease Research Center, University of Wisconsin School of Medicine and Public Health, University of Wisconsin-Madison, 600 Highland Ave J5/1 Mezzanine, Madison, WI USA; 8 Department of Pathology, Immunology and Laboratory Medicine, Rutgers New Jersey Medical School, Rutgers Biomedical and Health Sciences, Medical Science Building 185 South Orange Avenue, Newark, NJ USA

**Keywords:** Plasma p-tau, Amyloid-beta, Medial temporal lobe, Dynamic flexibility, Generalization, African Americans

## Abstract

**Background:**

Phosphorylated tau (p-tau) and amyloid beta (Aβ) in human plasma may provide an affordable and minimally invasive method to evaluate Alzheimer’s disease (AD) pathophysiology. The medial temporal lobe (MTL) is susceptible to changes in structural integrity that are indicative of the disease progression. Among healthy adults, higher dynamic network flexibility within the MTL was shown to mediate better generalization of prior learning, a measure which has been demonstrated to predict cognitive decline and neural changes in preclinical AD longitudinally. Recent developments in cognitive, neural, and blood-based biomarkers of AD risk that may correspond with MTL changes. However, there is no comprehensive study on how these generalization biomarkers, long-term memory, MTL dynamic network flexibility, and plasma biomarkers are interrelated. This study investigated (1) the relationship between long-term memory, generalization performance, and MTL dynamic network flexibility and (2) how plasma p-tau231, p-tau181, and Aβ42/Aβ40 influence generalization, long-term memory, and MTL dynamics in cognitively unimpaired older African Americans.

**Methods:**

148 participants (*Mean*_*age*_: *70.88*,*SD*_*age*_: *6.05*) were drawn from the ongoing longitudinal study, *Pathways to Healthy Aging in African Americans* conducted at Rutgers University–Newark. Cognition was evaluated with the Rutgers Acquired Equivalence Task (generalization task) and Rey Auditory Learning Test (RAVLT) delayed recall. MTL dynamic network connectivity was measured from functional Magnetic Resonance Imaging data. Plasma p-tau231, p-tau181, and Aβ42/Aβ40 were measured from blood samples.

**Results:**

There was a significant positive correlation between generalization performance and MTL Dynamic Network Flexibility (*t* = 3.372, *β* = 0.280, *p* < 0.001). There were significant negative correlations between generalization performance and plasma p-tau231 (*t* = -3.324, *β* = -0.265, *p* = 0.001) and p-tau181 (*t* = -2.408, *β* = -0.192, *p* = 0.017). A significant negative correlation was found between plasma p-tau231 and MTL Dynamic Network Flexibility (*t* = -2.825, *β* = -0.232, *p* = 0.005).

**Conclusions:**

Increased levels of p-tau231 are associated with impaired generalization abilities and reduced dynamic network flexibility within the MTL. Plasma p-tau231 may serve as a potential biomarker for assessing cognitive decline and neural changes in cognitively unimpaired older African Americans.

## Background

Alzheimer’s disease (AD) is a progressive neurodegenerative disease characterized by in its early stages the widespread neural deposition of amyloid-β (Aβ) plaques, followed by tau aggregation into neurofibrillary tangles in the regions such as entorhinal cortex which is part of Medial Temporal Lobe (MTL), and resulting in neurodegeneration, cognitive decline, and ultimately dementia [1–3]. Approximately 6.7 million Americans aged 65 and older (10.7%) have AD, and by 2060, the number is expected to nearly triple [4]. It is projected that the number of African Americans, aged 65 and older, affected by AD will double by 2030 [5]. Additionally, African American older adults are disproportionately affected by AD, exhibiting higher prevalence rates and earlier onset compared to their White counterparts [6, 7]. This increased risk is influenced by a combination of biological, genetic, and social factors, including a higher prevalence of coexisting cerebrovascular pathologies, such as hypertension and diabetes, which can interact with AD pathology [8]. These non-AD co-pathologies may contribute to structural and functional differences in brain regions [9]. Moreover, social and contextual factors—including socioeconomic disparities, reduced access to healthcare, and educational inequalities—further contribute to the elevated dementia risk among African Americans [8, 9]. One significant barrier to research participation among African Americans is the perceived invasiveness of certain procedures [10]. Although positron emission tomography (PET) imaging provides valuable biomarker information, it is often costly, time-intensive, and perceived as intrusive [11]. Blood-based biomarkers present a more cost-effective and accessible alternative, potentially increasing the willingness of African Americans and other underrepresented groups to engage in research studies [12, 13]. Despite the higher incidence and prevalence of AD among African Americans, this group continues to be underrepresented in AD research, especially in clinical trials.

The pathological processes underlying AD begin decades before the onset of clinical symptoms, starting with as an increase in amyloid plaque and tangle burden [14, 15]. Several in vivo biomarkers of neurofibrillary tangle development include PET and soluble phosphorylated tau (p-tau) levels in human cerebrospinal fluid (CSF), and more recently p-tau in blood plasma [16]. PET biomarkers have the advantage of providing spatial information on tracer binding throughout the brain but are invasive and expensive. To address this, blood-based biomarkers have emerged as a more accessible, noninvasive, and relatively inexpensive alternative [17]. Specifically, higher levels of plasma p-tau have been link to dementia development, shown to improve risk stratification for AD dementia and have demonstrated specific diagnostic abilities to distinguish AD from other neurodegenerative diseases [18]. Mechanistically, several subtypes of p-tau markers, which differ at various phosphorylation sites, such as p-tau181, p-tau217, and p-tau231, have been recently introduced as valuable markers of AD [19, 20]. In particular, plasma p-tau231 has emerged among the most sensitive biomarkers for detecting early stages of preclinical AD pathology [21]. While not as sensitive to preclinical AD pathology, plasma p-tau181 may differentiate AD from non-AD neurodegenerative diseases and predict the progression of AD dementia [22]. Overall, this post-translational modification of tau protein is implicated in the pathological processes of AD and serves as a critical marker for in vivo tau pathology. Elevated levels of p-tau in plasma and CSF correlate strongly with neurofibrillary tangles, a hallmark of AD pathology, thereby providing a non-invasive method to track tau-related neurodegeneration [23, 24]. Additionally, plasma Aβ has been used to assess amyloid pathology in AD [25]. While amyloid burden is upstream event in the AD pathological cascade [26], this deposition, particularly when evaluated alongside tau markers, may provide a more comprehensive view of disease progression [27–30]. Furthermore, Aβ42/Aβ40 ratio adjusts for inter-individual differences in the concentrations of the aggregation-prone Aβ42 peptide, making the ratio a more reliable indicator of Aβ plaque pathology compared with Aβ42 alone [31].

Complementary methods, such as resting-state functional magnetic resonance imaging (rs-fMRI) coupled with cognitive testing, may help index the functions disrupted by such AD-related pathologies. The rs-fMRI approach, in particular, may help characterize dynamic functional connections of the MTL [32]. Tau accumulation is first observed in the transentorhinal and entorhinal cortex, later spreading into adjacent association and unimodal cortices [33–35]. The entorhinal cortex, a component of the MTL memory system, is the gateway for information entering and leaving the hippocampal formation [36]. Therefore, MTL dynamics may be crucial to understand preclinical AD related pathological changes. MTL dynamic network flexibility refers to the ability of the MTL network to exhibit variable patterns of connectivity over time, as measured by rs-fMRI [37–39]. This approach highlights how connectivity within the MTL can dynamically change in response to different states of rest, reflecting the network’s adaptability and functional reorganization over time. MTL dynamic flexibility reflects the network’s ability to switch between different connectivity states, which is crucial for supporting various cognitive processes and maintaining flexibility in responding to new information [37, 40]. Older adults may show less variability in connectivity patterns, suggesting reduced adaptability and capacity for functional reorganization within the MTL network. This reduction in dynamic flexibility may be linked to the cognitive declines observed with age [41]. In the early stages of AD, changes in dynamic connectivity patterns in the MTL may serve as early indicators of AD pathology and may be associated with more pronounced cognitive deficits and progression of neurodegenerative changes [42]. These subtle neural changes in the preclinical stage may underlie subtle cognitive alterations.

AD becomes increasingly prevalent as individuals live longer worldwide, and there is a critical need for strategies to prevent or delay its onset. Early diagnosis of AD, even in the preclinical stage when symptoms are not yet present, could potentially lead to strategies to help individuals maintain their everyday functions [43]. Neuropsychological measures are used to detect early impairment and decline in preclinical AD [44]. However, traditional laboratory-based cognitive assessments can be expensive and time-consuming, or may lack sensitivity to early-stage changes [45]. Conversely, computational cognitive tests provide a more streamlined evaluation while maintaining or even enhancing sensitivity. Identifying novel markers that are sensitive to the subtle changes associated with preclinical AD can facilitate early detection and offer more options for preventing the disease from progressing [46].

Generalization—the cognitive ability to apply learned knowledge or skills to novel situations—is a critical aspect of adaptive behavior and cognitive flexibility [40, 47]. As individuals age, there is a notable decline in generalization capabilities. Research has shown that older adults may exhibit reduced flexibility in applying learned information to new contexts, which can manifest as difficulties in problem-solving and adapting to novel tasks [40]. Studies have demonstrated that individuals with preclinical AD, often identified through biomarkers and subtle cognitive changes, show impairments in the ability to generalize past experiences to new tasks [48, 49]. Generalization longitudinally predicts cognitive decline and neural changes in preclinical AD [50]. This decline in generalization is thought to be associated with age-related changes in brain structures and functions, particularly in the MTL and prefrontal cortex, which are crucial for integrating and applying information across different contexts [51]. Early computational models by Gluck and Myers demonstrated that by altering task demands, MTL circuits may have difficulties with generalization of prior learning, despite intact initial learning of associations between stimuli [52]. More recently, higher dynamic network flexibility within the MTL was shown to mediate better generalization of prior learning [37].

Currently, however, there is not a comprehensive understanding of how these cognitive (generalization) and neural biomarkers (MTL dynamic network flexibility) relate to validated blood-based biomarkers. No previous study has examined these markers of AD together as they relate to blood-based biomarkers nor within cognitively unimpaired older African Americans at risk for AD. As such, the purpose of this study was to investigate: (1) the relationship between long-term memory, generalization performance, and MTL dynamic network flexibility and (2) how plasma p-tau231, p-tau181, and Aβ42/Aβ40 influence the relationship between generalization, long-term memory, and MTL dynamics in cognitively unimpaired older African Americans.

## Methods

### Participants

Participants were drawn from the ongoing longitudinal study, *Pathways to Healthy Aging in African Americans* conducted at Rutgers University–Newark. The *Pathways* study enrolls African Americans aged 60 and older and investigates relationships between cognition, health, lifestyle, genetics, brain structure and function, and sociodemographic variables. The *Aging & Brain Health Alliance*, a university-community partnership founded in 2006 with a membership consisting of community members from senior centers, public and subsidized housing associations, religious institutions, health and wellness organizations, and other organizations that respond to the well-being of the residents of greater Newark, was utilized to recruit participants.

Participants who were aged 60 years or older, identified as black or African American; met the criteria of a Mini-Mental State Examination (MMSE) score of 24 or above, and had undergone a rs-fMRI scan were included in the study. Participants who had any neurodegenerative disorders, took medications commonly prescribed for the treatment of dementia (e.g. Namenda, Razadyne, Aricept), had learning disabilities, self-reported abusing alcohol and/or drugs excessively, had a medical procedure that required general anesthesia in the past three months; reported having of MRI contraindications (e.g., cardiac pacemaker, metallic stent, claustrophobia); had color blindness (as certain tasks relied on distinguishing colors for cues) and/or declined to undergo a blood draw were excluded from the study. The Rutgers University Institutional Review Board approved the study as ethical. All methods were performed in accordance with the ethical standards as established in the 1964 Declaration of Helsinki and its later amendments or comparable ethical standards.

### Procedure

Candidates were telephone screened to determine initial eligibility. After providing informed consent, participants who passed the in-person examinations for color blindness and mental status proceeded on to a full laboratory visit that consisted of cognitive assessments. Participants provided a blood sample for p-tau231, p-tau181, and Aβ42/Aβ40 levels and returned for a neuroimaging session within two weeks of the initial laboratory visit.

## Measures

### Rutgers acquired equivalence task

Rutgers Acquired Equivalence Task was developed by the senior author (Gluck) and colleagues at Rutgers University-Newark. Generalization, the ability to apply previously learned rules to new situations and contexts, is subserved by the MTL [53–56]. Generalization trials require the adaptive application of associative knowledge, which involves the integrative encoding of information and its recombination during the retrieval process. Mnemonic flexibility, which is essential for successful generalization, relies heavily on the structural and functional integrity of the MTL [57]. Research indicates that individuals with MTL alterations linked to prodromal AD exhibit intact learning but show significant deficits in generalization performance [54, 55, 58, 59]. In this task, participants learn six pairs of stimulus-response relations in the acquisition phase of the task, stimuli are presented and responses are collected using laptop computer. Antecedent stimuli are drawings of four faces (2 women faces, 2 men faces). The consequents are drawings of red, orange, purple and pink fish. For each participant, faces and fish were randomly assigned as antecedent and consequent stimuli. The selected fish drawing is circled and corrective feedback is given (Fig. 2). The left-right order of the fish drawings is randomized across subjects. There are three phases in the acquisition phase (Table 1). The participant is not informed that a new phase has started and is not made aware of the existence of new associations. The transfer phase consists of 48 trials, 12 of which are new associations to test learned equivalence and 36 of which are old associations trained in the acquisition phase. Dependent measures are the average number of errors in the acquisition phase and the proportion of incorrect responses in the transfer phase [59].

In Stage 1 of the experimental protocol, participants engage in the acquisition of knowledge pertaining to the initial two pairings involving distinct individuals (A and B), and specific fish stimuli (X and Y). Participants become linked to the same fish stimuli in Stage 2, showing stimulus equivalence. Subsequently, in Stage 3, new consequents are introduced into the learning process. During the transfer phase, participants are subjected to testing not only on the associations cultivated in Stages 1 to 3 but also on entirely new associations that were not part of the initial learning in these stages but have arisen as a result of stimulus equivalence. This phase of stimulus generalization is closely linked with the MTL, while the learning occurring in Stages 1 to 3 is predominantly associated with the basal ganglia [59].

The task involves a sequence of trials wherein participants are presented with one face image alongside two fish stimuli, all concurrently displayed. Below this arrangement, participants are provided with a directive, instructing them to select either the left or right fish by using the corresponding key label for input. Initially, participants are required to make a random selection. Subsequent to their choice, the selected fish is circled, and immediate feedback is presented in the form of either “Correct” or “Incorrect.” This feedback, in conjunction with the face image, the two fish stimuli, and the encirclement, remains visible on the screen for a duration of 1 s. Following this, the screen undergoes a 1-second intertrial interval with a blank display prior to the initiation of the subsequent trial, featuring a novel face image and a new pair of fish stimuli (Table 1).

To calculate the accuracy score in the Rutgers Acquired Equivalence Task, each subject’s performance is assessed based on their correct responses during the acquisition and transfer phases. The accuracy score is the percentage of correct responses relative to the total number of trials. This can be calculated by dividing the number of correct trials by the total trials attempted and then multiplying by 100 to express the value as a percentage. Higher scores indicate better performance. Automated scoring tools were used to streamline and ensure consistency. For publicly available codes please see https://github.com/Aging-and-Brain-Health-Alliance/GluckLab.

**Table 1:**
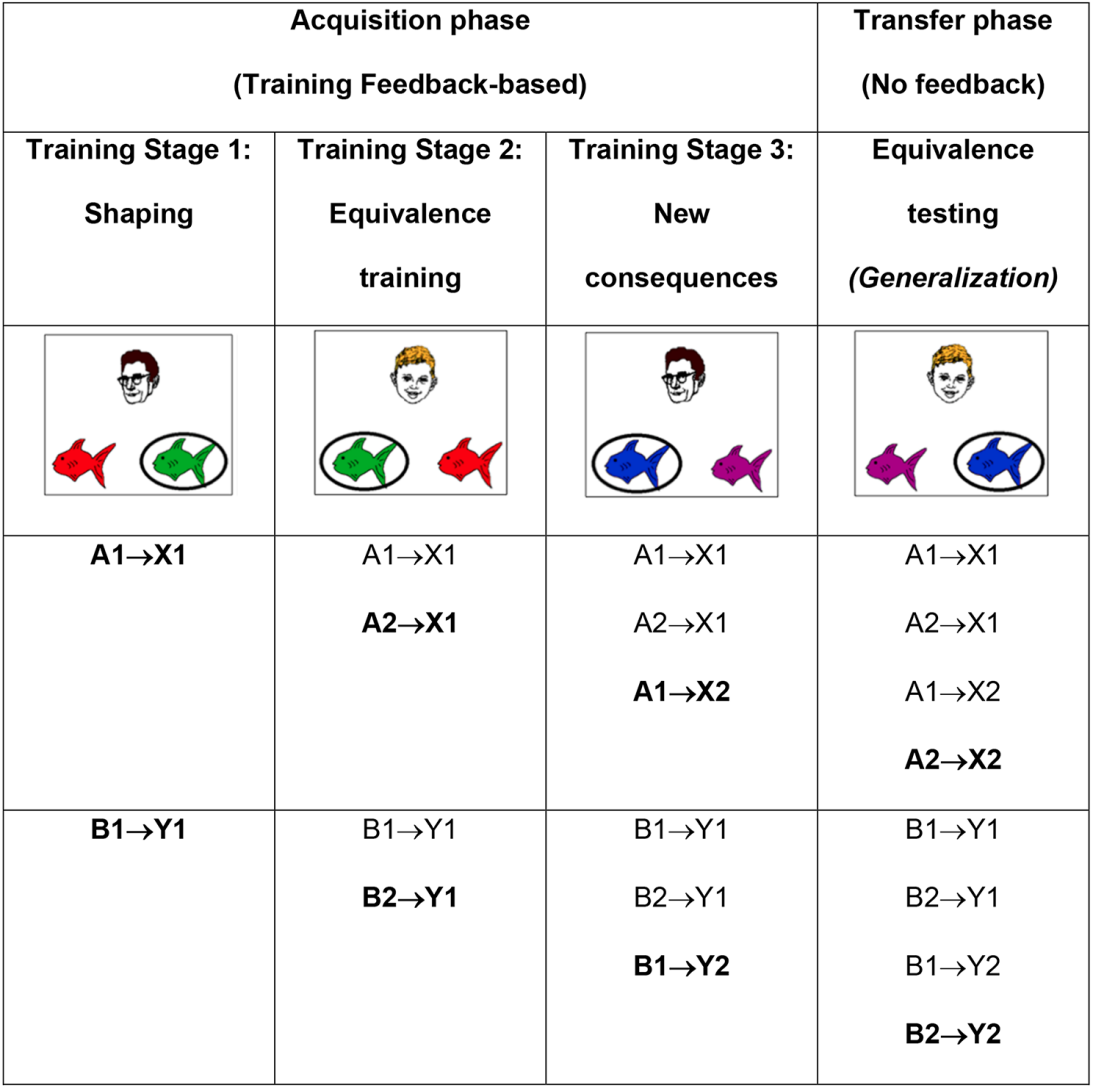
The acquired equivalence paradigm. During training stage 1, participants learn the first two associations between different faces (A1, B1) and fishes (X1, Y1). During training stage 2, different faces (A2, B2) are associated with the same fish, whereas during stage 3, new fishes (X2, Y2) are added. During the test phase, participants are tested on retention of the associations learned in training stages 1–3, and also on generalization to new pairings of faces and fishes (i. e., A2 → X2, B2 → Y2). Retention and generalization pairs were interleaved randomly during the test phase.

### Rey auditory learning test

The Rey Auditory Verbal Learning Test (RAVLT) is a widely recognized neuropsychological assessment designed to evaluate episodic memory, with particular sensitivity to verbal memory impairments commonly seen in neurological conditions such as AD. The RAVLT involves the sequential presentation of a 15-word list across five trials, during which the examiner reads the words aloud, and the participant is asked to recall as many words as possible immediately after each trial (Trials 1–5). Following these trials, a second, interference list (List B) of 15 new words is introduced, and the participant is again asked to recall the words. Subsequently, in Trial 6, the participant is prompted to recall the original list without further exposure. After a 20-minute delay, during which other cognitive tasks are administered, the participant is asked to recall the original list once more (delayed recall).

The present study utilized the RAVLT recall score. It was selected for it’s ability to capture distinct facet of episodic memory—memory retention over time (RAVLT Percent Forgetting)— which is critical in AD pathology. Previous studies have demonstrated significant correlations between the RAVLT delayed recall and AD progression, underscoring it’s relevance in our investigation [60–62]. Higher delayed recall scores indicate better episodic memory performance.

### Blood sample collection and plasma analysis

Blood samples were collected by a phlebotomist at the Clinical Research Unit at the Rutgers New Jersey Medical School using 6–10 mL EDTA tubes with all biosafety protocols in place. Samples were brought to the Fitzgerald-Bocarsly lab at NJMS, centrifuged within one hour and plasma was collected and aliquoted into 0.5 mL polypropylene tubes with screw caps, then directly frozen at -80 °C. One 0.5 mL aliquot of EDTA plasma per participant were transported on dry ice to the Clinical Neurochemistry Laboratory at the University of Gothenburg for analyses. Plasma p-tau231, p-tau181, and Aβ42/Aβ40 ratio (pg/mL) were measured using Single molecule array (Simoa) assays on an HD-X analyzer (Quanterix), as previously described [20].

### MRI data acquisition

Participants in the study underwent magnetic resonance imaging (MRI) procedures at the Rutgers University Brain Imaging Center (RUBIC) at Rutgers University-Newark through a 3T Siemens TRIO with a 32-channel Multiband parallel encoding coil. Each participant underwent a high-resolution 3D magnetization-prepared rapid gradient echo (MP-RAGE) structural scan and resting-state MRI in the sagittal plane. The scanning parameters were configured to attain optimal results: a repetition time (TR) of 1900 milliseconds, an echo time (TE) of 2.52 milliseconds, a precise 9-degree flip angle, 176 contiguous slices (without any inter-slice gap), voxel dimensions of 1.0 × 1.0 × 1.2 mm, and a field of view (FOV) measuring 270 × 254 × 212. This careful calibration led to a total acquisition time of 9 min, ensuring high-quality data collection. High-resolution Multiband echo-planar imaging techniques were configured with a FOV spanning 208 × 208 × 125, a TR of 664 milliseconds, an TE of 30 milliseconds, a 30-degree flip angle, an isotropic resolution of 1.8 mm with no interslice gap, and a Multiband acceleration factor of 5. The imaging protocol encompassed 45 axial slices, acquired in a simultaneous multiband parallel imaging, which facilitated the acquisition of high-resolution functional images while curtailing acquisition time and minimizing susceptibility-induced distortions. Furthermore, the high temporal efficiency demonstrated by this methodology conferred greater statistical power to the study’s analytical framework [63].

### fMRI data analysis

#### Preprocessing

The Analysis of Functional NeuroImages (AFNI) software was conducted to preprocess and analyze all neuroimaging data on a Linux platform. The standard afni_proc.py pipeline was predominantly utilized for data processing. The data preprocessing pipeline employed in this study adhered to standardized procedures for robust analyses. Initially, the functional data underwent several essential preprocessing steps. Outliers were mitigated through despiking (3dDespike), temporal alignment was executed via slice timing correction (3dtshift), and precise anatomical registration was achieved with MP-RAGE images using the align_epi_anat.py tool. Subsequently, motion artifacts were diligently corrected using 3dvolreg, followed by spatial smoothing with a Gaussian FWHM kernel to attain a 2 mm isotropic resolution (3dmerge). For improved data quality, an automatic brain mask (3dautomask) was applied to exclude extraneous voxels outside the brain. To ensure data integrity, a custom script was implemented to exclude trials exhibiting motion exceeding 0.3 mm from the time series, thereby enhancing the reliability of our analyses. Moreover, we incorporated a signal regression approach to address motion and scanner-related noise [64]. ANATICOR, which uses local white matter and ventricular signal estimations to the neighboring gray matter voxels, was used to regress out signal fluctuations [65]. Functional scans were registered to each subject’s skull-stripped MP-RAGE image using the align_epi_anat.py tool. Final voxel time courses were estimated using univariate regression (3dDeconvolve), which incorporated nuisance variables for the effects of six motion parameters (pitch, roll, and yaw; x, y, and z frame displacement) and linear scanner drift. To achieve a common anatomical reference frame across participants, the Advanced Normalization Tools (ANTs) were utilized, employing a diffeomorphic nonlinear registration algorithm (SyN) to warp each participant’s structural scan into an in-house high-resolution 0.65 mm isotropic template [66]. The coplanar functional data, derived from the aforementioned regression, were processed using these transformation parameters to align them with the custom template, facilitating both individual and group-level analyses.

#### Dynamic network construction

The investigation of dynamic functional connectivity in the MTL encompassed cortical and hippocampal subfields (subiculum, CA1, and DG/CA3), as well as the perirhinal cortex (PRC), parahippocampal cortex (PHC), posteromedial entorhinal cortex (pMEC), and anterolateral entorhinal cortex (aLEC). The perirhinal and parahippocampal regions, respectively, provide input to the lateral and medial entorhinal cortices, which ultimately supply input to the hippocampus. Evidence suggests an anterolateral to posteromedial functional division in the human EC [67, 68]. According to prior studies, there are functional distinctions between the lateral and medial EC [69]. This study consequently regarded the pmEC and alEC as separate regions of interest (ROIs) within the MTL network. For each ROI (3dmaskave), the average time series of 812 time points was derived. The time series were subsequently separated into sub-blocks of 50-time points (33 s), yielding a total of 16 time windows, in order to evaluate the dynamic connectivity between ROIs. The initial six and the last six time points were omitted. The time window’s duration was set to be long enough to allow for an accurate assessment of correlations throughout frequencies present in the wavelet band of interest (0.06–0.12 Hz), yet brief enough to allow for a fine-grained measurement of temporal evolution over the whole session [70]. According to previous studies, connectivity was then determined for each of the 16 sub-blocks as the magnitude squared spectral coherence between each pair of ROIs to measure modularity over time frames [71–73]. Subject-specific 7 × 7 × 16 connectivity matrices with coherence values ranging from 0 to 1 were constructed for 7 ROIs and 16-time windows. Coherence was used to quantify frequency-specific linear correlations between time series, which has been demonstrated to be effective in the context of fMRI data [74]. A multilayer network technique was utilized to investigate changes in functional brain network architecture. In this methodology, each layer of the network was built up from connectivity matrices representing distinct time windows, allowing for the analysis of temporal changes in network properties [75]. For each participant, multilayer networks were constructed by linking nodes in the connectivity matrix of one time window to their corresponding nodes in the matrices of adjacent time windows [76]. This inter-layer connectivity facilitated the creation of a time-dependent network structure, wherein each node was connected to its equivalent in previous and subsequent time slices. Consequently, this representation enabled the identification of densely interconnected subgroups, referred to as communities or modules, whose identities were consistently tracked across temporal frames. This approach provides a robust framework for examining dynamic changes in brain connectivity and network modularity over time [72, 77].

#### Dynamic community detection

A Louvain-like locally greedy community detection algorithm for optimizing multilayer modularity was used to partition each multilayer network into temporally connected modules [77]. A community assignment for each node and time frame, representing the module allegiance, was generated by optimizing multilayer modularity. The flexibility of each node was defined as the extent to which it altered its module allegiance throughout the set of time frames represented by the multilayer network to enable it to measure changes in the composition of communities across time [71]. Therefore, flexibility was calculated for each of our 7 ROIs (PRC, PHC, pMEC, aLEC, subiculum, CA1, DG/CA3) as the number of times a node indicated a change in community assignment, normalized by the total number of changes that may occur. The dynamic flexibility of the MTL network was then computed as the mean flexibility over all nodes.

### Statistical analysis

IBM SPSS (Statistical Package for Social Science) version 29.0 was used for statistical analysis. Summary statistics and histograms were generated to explore the distributions of data, providing insights into central tendencies and variability. Outliers were identified through visual inspection and statistical criteria; specifically, values exceeding two times the interquartile range (IQR) from first and third quartiles were flagged as potential outliers. Additionally, standardized residuals exceeding ± 2.0 were evaluated. However, no extreme outliers were found to significantly affect the results, therefore, all data points were retained for analysis. A series of linear regression analyses were conducted to examine: (1) the relationship between long-term memory, generalization performance, and MTL dynamic network flexibility, and (2) the influence of plasma p-tau231, p-tau181 and Aβ42/Aβ40 on generalization performance, long-term memory, and MTL dynamics in cognitively unimpaired older African Americans. Age, sex, and education level (*and Acquisition score was only used for Rutgers Acquired Equvalence Task*) were included as covariates to control for potential condounfing factors. Prior to conducting the regression analyses, assumptions of linearity, normality, homoscedasticity, and independence were verified. The overall model fit was assessed using R² values and F-statistics.The significance value was accepted as *p* < 0.05.

### Power analysis

The sample size was determined using the G*power sample size calculator [78]. The sample size was calculated as 71 subjects using “Correlation: Point biserial model” design with two tails and a power of 95% (*α =* 0.05, *β =* 0.95, *t =* 1.994) and effect size of 0.40.

## Results

### Demographics

Demographics of the 148 participants (98 women) are shown in Table 2. Participants were ages 60 to 88 years old and completed on average, fourteen years of education.

**Table 2:** Distribution of demographic data

(*N* = 148)		Avg ± SD
Age (years)		70.88 ± 6.05
Gender (n (%))	Women	98 (66.21)
	Men	50 (33.79)
Education (years)		13.90 ± 2.47
MMSE		26.98 ± 2.14
MTL Dynamic Network Flexibility		0.34 ± 0.26
Rutgers Acquired Equivalence Task Accuracy		0.57 ± 0.24
RAVLT Delayed Recall		7.93 ± 3.60
Plasma p-tau231 (pg/mL)		20.39 ± 11.99
Plasma p-tau181 (pg/mL)		18.68 ± 9.49
Aβ42/Aβ40 ratio (pg/mL)		0.06 ± 0.00

MMSE = Mini-Mental State Examination; MTL = Medial Temporal Lobe; RAVLT = Rey Auditory Learning Test, Aβ = Amyloid Beta, Avg = Average; SD = Standard Deviation

### Relationship between cognitive measures and MTL dynamic network flexibility

**Fig. 1:**
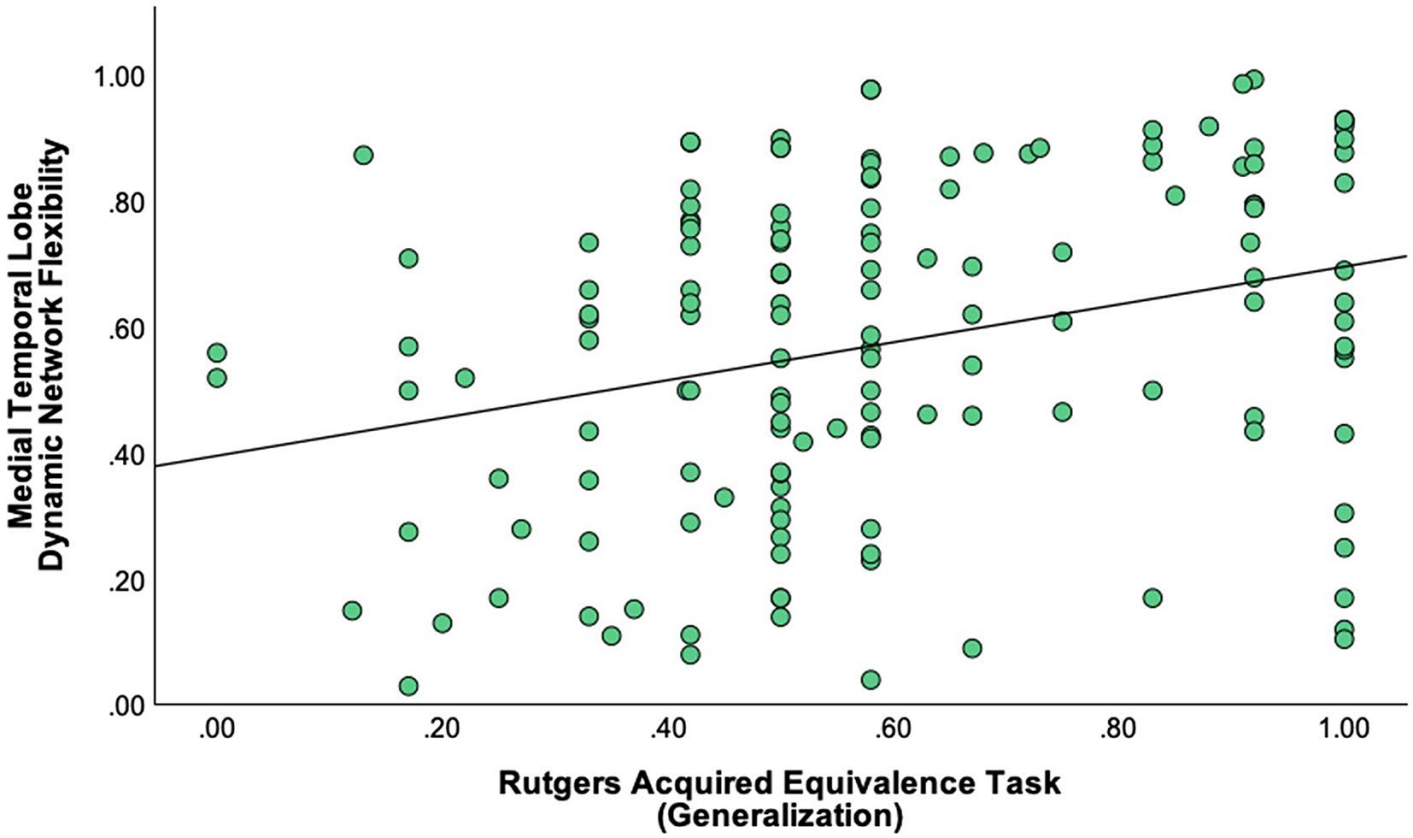
Better generalization performance is associated with higher Medical Temporal Lobe Dynamic Network Flexibility

A statistically significant positive correlation was found between Rutgers Acquired Equivalence Task and MTL Dynamic Network Flexibility with generalization accuracy score emerging as a significant predictor (*t* = 3.372, *β* = 0.280, *p* < 0.001). The overall regression model was significant (*F*_*(5, 141)*_ = 5.430, *p* < 0.001), accounting for 16.1% of the variance in MTL Dynamic Network Flexibility (*R²* = 0.161), with a model strength of *R* = 0.402 (Fig. 1). There was no statistically significant correlation between MTL Dynamic Network Flexibility and RAVLT Delayed Recall (*t* = 0.803, *β* = 0.067, *p* = 0.423).

### Relationship between plasma biomarkers and generalization performance

**Fig. 2:**
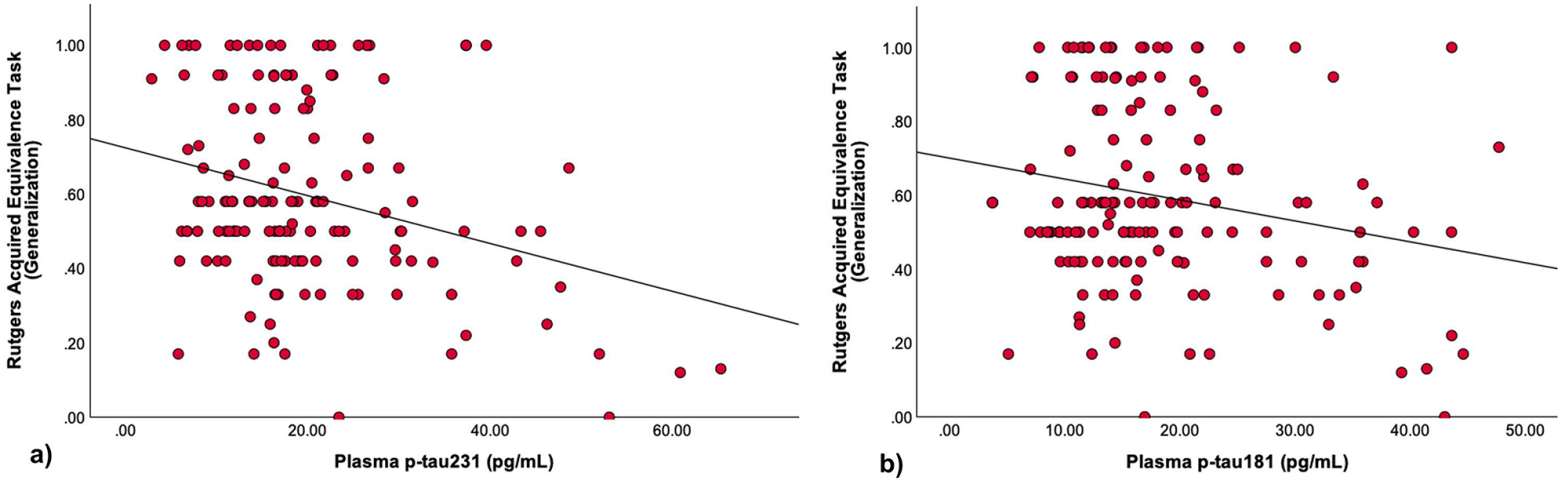
Lower generalization performance is associated with **a**) Elevated plasma p-tau231 **b**) Elevated plasma p-tau181

There was a statistically significant negative correlation between plasma p-tau231 levels and Rutgers Acquired Equivalence Task with plasma p-tau231 levels emerging as a significant predictor (*t* = -3.324, *β* = -0.265, *p* = 0.001). The overall regression model was significant (*F*_*(5, 141)*_ = 7.064, *p* < 0.001), accounting for 20% of the variance in generalization performance (*R²* = 0.200), with a model strength of *R* = 0.448 (Fig. 2a). A statistically significant negative correlation was observed between plasma p-tau181 levels and generalization performance with plasma p-tau181 levels emerging as a significant predictor (*t* = -2.408, *β* = -0.192, *p* = 0.017). The overall regression model was significant (*F*_*(5, 141)*_ = 5.846, *p* < 0.001), accounting for 17.2% of the variance in generalization performance (*R²* = 0.172), with a model strength of *R* = 0.414 (Fig. 2b). No statistically significant correlation was found between generalization performance and Aβ42/Aβ40 ratio (*t* = − 0.352, *β* = − 0.028, *p* = 0.726).

### Relationship between plasma biomarkers and RAVLT delayed recall

There was no statistically significant correlation was found between RAVLT delayed recall and p-tau231 (*t* = -1.350, *β* = − 0.113, *p* = 0.179), p-tau181 (*t* = -1.399, *β* = − 0.114, *p* = 0.164), and Aβ42/Aβ40 ratio (*t* = − 0.228, *β* = − 0.018, *p* = 0.820).

### Relationship between plasma biomarkers and MTL dynamic network flexibility

**Fig. 3:**
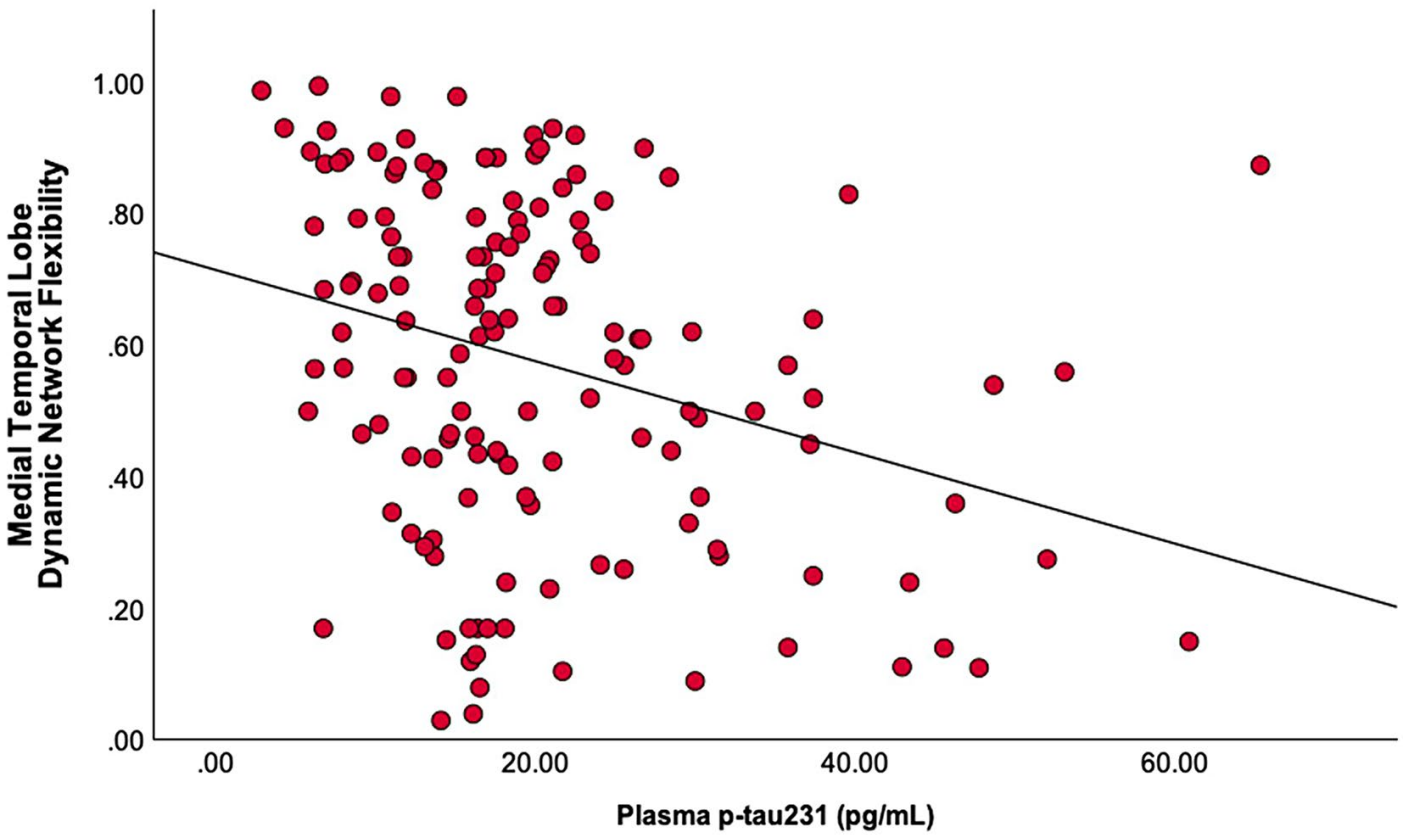
Elevated plasma p-tau 231 is associated with reduced Medical Temporal Lobe Dynamic Network Flexibility

A statistically significant negative correlation between plasma p-tau231 levels and MTL Dynamic Network Flexibility with plasma p-tau231 levels emerging as a significant predictor (*t* = -2.825, *β* = -0.232, *p* = 0.005). The overall regression model was significant (*F*_*(4, 143)*_ = 6.051, *p* < 0.001), accounting for 14.5% of the variance in MTL Dynamic Network Flexibility (*R²* = 0.145), with a model strength of *R* = 0.380 (Fig. 3). There was no statistically significant correlation between MTL Dynamic Network Flexibility and p-tau181 (*t* = -1.338, *β* = -0.110, *p* = 0.183), and Aβ42/Aβ40 ratio (*t* = − 0.464, *β* = − 0.038, *p* = 0.643).

## Discussion

In this study, we found that [1] better generalization performance was associated with higher MTL dynamic network flexibility [2], elevated plasma p-tau231 and p-tau181 levels were associated with reduced generalization performance, and [3] elevated plasma p-tau 231 levels was associated with reduced MTL dynamic network flexibility in a cohort of cognitively unimpaired older African Americans. We conclude that elevated levels of plasma biomarkers may reflect underlying neurodegenerative processes that impair cognitive function as captured by novel measures, and underscores the potential impact of plasma p-tau pathology on neural dynamics within the MTL among cognitively unimpaired older African Americans.

In preclinical AD, memory decline may manifest subtly, as individuals in this stage often remain cognitively unimpaired according to standard clinical criteria [79]. However, memory deficits can emerge in tasks like delayed recall [80]. The delayed recall score of the RAVLT captures the ability to retain and retrieve information after a delay, which is often compromised in individuals with AD-related pathology [81]. Generalization serves as a critical cognitive function that is affected by aging, neurodegeneration, and early stages of AD. Understanding how generalization is impacted in these contexts provides valuable insights into the progression of cognitive decline and highlights the potential for using generalization deficits as a cognitive biomarker for early detection and monitoring of neurodegenerative diseases. Generalization is unable to occur without an accurate neural representation of stimulus interactions [82]. As such, Sinha and colleagues (2021) investigated the relationship between generalization and the MTL, a major site of neuroplasticity [37]. They concluded that the MTL is sensitive to generalization and is one of the earliest brain regions impacted by AD. In this study, we observed a positive relationship between generalization accuracy performance and MTL Dynamic Network Flexibility, suggesting that greater network adaptability in the MTL may support cognitive flexibility and generalization abilities. Interestingly, there was no relationship between RAVLT delayed recall and Dynamic Network Flexibility. This lack of association may indicate that while MTL network flexibility plays a role in tasks requiring cognitive flexibility, it may not be as strongly linked to long-term memory consolidation, as measured by the RAVLT delayed recall.

Delayed recall, a critical component of long-term episodic memory, reflects an individual’s ability to retrieve previously learned information after a period of time [83]. Older African Americans obtain significantly lower scores on measures of verbal and nonverbal learning and memory compared to White counterparts [84]. On the other hand, Petok et al. (2018) observed that generalization deficits on the Rutgers Acquired Equivalence Task appear years before overt symptoms of cognitive decline, suggesting that generalization tests could more accurately capture subtle differences than standard diagnostic methods such as a neuropsychological assessments [48]. In the context of aging, increased p-tau and Aβ levels are associated with cognitive impairment and accelerated cognitive decline [85]. In light of the emergence of plasma p-tau231, p-tau181, and Aβ42/Aβ40 as sensitive biomarkers for AD risk, linking plasma biomarkers and generalization performance can play a critical role in detecting AD at the preclinical stage. In the present study, we examined the influence of plasma biomarkers on generalization performance and RAVLT delayed recall. We found negative relationships between plasma biomarkers (p-tau231 and p-tau181) and generalization performance. However, there was no relationship between plasma Aβ42/Aβ40 and generalization performance. Research has demonstrated that higher plasma p-tau levels is linked with reduced cognitive performance in domains such as episodic memory and executive function, reflecting its role in the early detection of AD pathology before clinical symptoms become apparent [86]. However, we observed no relationship between plasma biomarkers and RAVLT delayed recall, as a marker of episodic memory dysfunction. A recent study emphasized that plasma p-tau231 may be a more robust indicator of Aβ and tau pathologies in preclinical disease than p-tau181 [19]. In parallel with this result, we observed that the relationship between plasma p-tau231 levels and generalization performance was stronger compared to the relationship with plasma p-tau181 levels. This differential effect may suggest that p-tau231 is a more sensitive marker of cognitive flexibility, particularly in processes related to generalization, in cognitively unimpaired older African Americans. This result underscores the potential of plasma p-tau231 as a biomarker to detect subtle cognitive changes prior to the clinical symptoms of AD.

Recent advances in plasma-based assays allow for the detection of tau phosphorylation at different residues, such as p-tau231 and p-tau181, offering insight into tau pathology progression, and were thus worth investigating in relation to measures of generalization performance and MTL dynamic network flexibility [87]. Plasma p-tau231 has emerged as an early marker of tau deposition in the brain, particularly in regions such as the entorhinal cortex, which is closely linked to MTL function [20]. Higher levels of p-tau231 in cognitively unimpaired individuals have been associated with greater MTL atrophy, a hallmark of AD progression [88]. This is especially relevant for African American populations, who are understudied in AD research, yet disproportionately affected by the disease. Plasma p-tau181, a well-established marker for AD-related neurodegeneration, is also significantly elevated in individuals at risk for AD [89]. Plasma p-tau181 may reflects tau pathology spread from the hippocampal regions into broader cortical areas [90]. In contrast, plasma Aβ42/Aβ40 ratio has been used to assess amyloid pathology in AD [91]. While amyloid plaques have been traditionally viewed as upstream events in the AD pathological cascade, recent studies have suggested that amyloid deposition, particularly when evaluated alongside tau markers, may provide a more comprehensive view of disease progression [29, 46, 92]. We found a significant negative relationship between plasma p-tau231 and MTL dynamic network flexibility, which may indicate that elevated p-tau231 interferes with the neural adaptability needed for generalization, a key cognitive process for learning and memory. In line with these findings, our results show a negative association between plasma p-tau181 and generalization performance, further implicating tau pathology in cognitive decline during the preclinical stages of AD. Though the current study did not find significant direct effects of Aβ42/Aβ40 on generalization or MTL dynamics, these ratios remain a critical factor when evaluating overall AD risk, particularly as amyloid and tau pathologies interact across the continuum of disease progression. Notably, these results highlight the possibility that p-tau181 and p-tau231 capture distinct aspects of tau-related neurodegeneration, with p-tau231 potentially reflecting earlier or more localized tau accumulation in the MTL, and p-tau181 capturing broader cortical involvement. These findings underscore the importance of early plasma biomarkers in detecting AD risk, particularly in populations like African Americans who are at elevated risk but often underrepresented in research. Plasma biomarkers such as p-tau231 and p-tau181 not only offer a minimally invasive alternative to PET imaging to track AD-related changes in the brain but may also serve as key indicators of cognitive decline related to MTL dysfunction. Recent advances in the development of AD blood tests provide a simple and cost-effective way to detect AD pathology and have the potential to increase access of minoritized groups to AD biomarker testing, whereas PET scans are expensive, involve exposure to low levels of radiation, and available only at specialized medical centers [93, 94]. Future studies are recommended to explore the interaction between plasma amyloid and plasma tau biomarkers in modulating MTL dynamics and generalization performance, expanding on the mechanistic underpinnings of AD progression.

Despite the increased risk of AD, African Americans have been historically underrepresented in biomarker, cognitive, and neuroimaging research on AD, limiting our understanding of disease mechanisms in this population [95]. Investigating plasma biomarkers and their association with cognitive performance and MTL network dynamics in African Americans provides crucial insights into early pathological processes that may underlie cognitive decline. These findings are particularly relevant given the emerging evidence that tau pathology may manifest differently across racial and ethnic groups [96]. By focusing on a cohort of cognitively unimpaired older African Americans, this study addresses critical gaps in the literature, offering valuable data on the early neural and cognitive correlates of AD in this population. Such insights could inform the development of more targeted diagnostic and therapeutic approaches, ultimately contributing to efforts to reduce health disparities in AD outcomes [97].

Our study is not without limitations. First, female participants comprised the majority of the analytic sample. Future studies should recruit higher proportions of males in order to test for sex differences in the interrelationships between plasma biomarkers, generalization performance, long-term memory, and MTL dynamic network flexibility. Second, we did not include genetic biomarkers, such as APOE and/or ABCA7 into our analysis. Future studies including genetic biomarkers into the analysis are recommended to more comprehensively assess AD risk. Furthermore, the underrepresentation of older African Americans in broader AD research hinders ability to most adequately situate these research findings for the purposes for addressing health disparities immediately. There is an imperative need to more comprehensively understand of AD related biomarkers within this population to guide the development of targeted treatments and potential interventions that address the needs of diverse older adults.

## Conclusions

AD will continue to present a tremendous challenge, especially among African Americans who bear a disproportionate burden of the disease. There is a need for a better understanding of early changes associated with preclinical AD to facilitate early detection and intervention. This study highlights (1) the importance of neural plasticity in optimizing learning and memory, (2) that elevated tau biomarkers may reflect neurodegenerative processes that impair cognitive function, and (3) the potential impact of tau pathology on neural dynamics in MTL among cognitively unimpaired older African Americans. Future studies are recommended to investigate targeted intervention strategies aimed at mitigating plasma tau-induced neurodegenerative changes in at-risk populations such as African Americans for early diagnosis of AD.

## Data Availability

No datasets were generated or analysed during the current study.

## Abbreviations

Aβ: Amyloid Beta
ABCA7: Adenosine Triphosphate–Binding Cassette Subfamily A Member 7
AD: Alzheimer’s Disease
AFNI: Analysis of Functional NeuroImages
aLEC: Anterolateral Entorhinal Cortex
ANTs: Advanced Normalization Tools
APOE: ApolipoproteinE
AVG: Average
CA1, DG/CA3: Subfields of the Hippocampus (Cornu Ammonis 1, Dentate Gyrus/Cornu Ammonis 3)
CSF: Cerebrospinal Fluid
EDTA: Ethylenediaminetetraacetic Acid
FOV: Field of View
FWHM: Full Width at Half Maximum
IBM SPSS: (Statistical Package for Social Science)
MTL: Medial Temporal Lobe
MP-RAGE: Magnetization-Prepared Rapid Acquisition with Gradient Echo
MRI: Magnetic Resonance Imaging
MMSE: Mini-Mental State Examination
NJMS: New Jersey Medical School
PET: Positron Emission Tomography
PHC: Parahippocampal Cortex
pMEC: Posteromedial Entorhinal Cortex
p-tau: Phosphorylated Tau
PRC: Perirhinal Cortex
ROI: Region of Interest
RUBIC: Rutgers University Brain Imaging Center
rs-fMRI: Resting-State Functional Magnetic Resonance Imaging
SD: Standard Deviation
Simoa: Single Molecule Array
SyN: Symmetric Normalization
TE: Echo Time
TR: Repetition Time
